# The role of perceived well-being in the family, school and peer context in adolescents’ subjective health complaints: evidence from a Greek cross-sectional study

**DOI:** 10.1186/1751-0759-7-17

**Published:** 2013-11-28

**Authors:** Dimitra Petanidou, Evangelie Daskagianni, Christine Dimitrakaki, Gerasimos Kolaitis, Yannis Tountas

**Affiliations:** 1 Centre for Health Services Research, Department of Hygiene, Epidemiology and Medical Statistics, Athens University Medical School, 25 Alexandroupoleos str., Athens 11527, Greece; 2 Institute of Social and Preventive Medicine, 25 Alexandroupoleos str., Athens 11527, Greece; 3 Department of Child Psychiatry, Athens University Medical School, “Aghia Sophia” Children’s Hospital, Greece. Thivon and Papadiamantopoulou, Athens 11527, Greece

**Keywords:** Subjective Health Complaints (SHC), Adolescence, Family, School, Peers, Well-being, Psychosomatic health, Subjective perceptions, Subjective economic status

## Abstract

**Background:**

During adolescence children are usually confronted with an expanding social arena. Apart from families, schools and neighbourhoods, peers, classmates, teachers, and other adult figures gain increasing importance for adolescent socio-emotional adjustment. The aim of the present study was to investigate the extent to which Greek adolescents’ perceived well-being in three main social contexts (family, school and peers) predicted self-reported Subjective Health Complaints.

**Methods:**

Questionnaires were administered to a Greek nation-wide, random, school-based sample of children aged 12–18 years in 2003. Data from 1.087 adolescents were analyzed. A hierarchical regression model with Subjective Health Complaints as the outcome variable was employed in order to i) control for the effects of previously well-established demographic factors (sex, age and subjective economic status) and ii) to identify the unique proportion of variance attributed to each context. Bivariate correlations and multicollinearity were also explored.

**Results:**

As hypothesized, adolescents’ perceived well-being in each of the three social contexts appeared to hold unique proportions of variance in self-reported Subjective Health Complaints, after controlling for the effects of sex, age and subjective economic status. In addition, our final model confirmed that the explained variance in SHC was accumulated from each social context studied. The regression models were statistically significant and explained a total of approximately 24% of the variance in Subjective Health Complaints.

**Conclusions:**

Our study delineated the unique and cumulative contributions of adolescents’ perceived well-being in the family, school and peer setting in the explanation of Subjective Health Complaints. Apart from families, schools, teachers and peers appear to have a salient role in adolescent psychosomatic adjustment. A thorough understanding of the relationship between adolescents’ Subjective Health Complaints and perceived well-being in their social contexts could not only lead to more effective tailored initiatives, but also to promote a multi- and inter-disciplinary culture in adolescent psychosomatic health.

## Background

During adolescence, the child transits from a circumscribed, family-centered milieu to a broader social arena, where the number of persons and contexts with a potential key role in shaping children’s behaviours, ambitions and resources is multiplied. The expansion of adolescents’ social circle holds various challenges, such as increased social pressure and responsibilities [1,2]. These challenges may be experienced as potential sources of stress with adverse effects on adolescent health, i.e. internalizing disorders and deficits in psychosomatic health [3].

Symptoms of elusive aetiology –such as headache, stomachache, nervousness and irritability broadly labeled as “Subjective Health Complaints” (SHC) – are often reported in adolescence. They have been related to limitations in daily functioning -such as school absenteeism- and have been considered as indicators of psychosocial maladjustment with implications, such as high medication and health services use, that may well extend to adult years [4]. Due to ample evidence linking SHC with independent and cumulative stressors in adolescents’ social contexts, scholars and clinicians consent that SHC should be examined and interpreted through the lens of the complex relational web that is formulated during adolescent years [2,5-7].

Among social contexts, family has been underlined to hold a profound role in psychosomatic symptoms in childhood and adolescence. Numerous studies have concluded that a supportive parent–child relationship and a low conflict family atmosphere are inversely related to SHC [2,8,9]. As the child moves throughout adolescence, he/she is usually called to attend long, compulsory school curriculums and is gradually inclined to spend an increasing amount of time with peers. In paediatric psychosomatic health literature, there is ample evidence documenting associations between adolescents’ SHC with several aspects of school life that may be experienced as potential sources of stress. Workload pressure -referring to both too high/low schoolwork- [2,7,9-13], a negative school climate [2,9,14], poor relationships with teachers [10,14-17] and classmates [7,8,14,17] have been considered sources of school distress and have been consistently associated with increased levels of SHC. Similarly, lack of supportive social affiliations or of close social ties with peers has been reported to thwart adolescent psychosomatic adjustment [5,8,18].

A limited number of studies has been dedicated in exploring the contributions of two [5,9,14,19] or three [1,16,20-23] social spheres of adolescents’ life in self-reported SHC, focusing on different facets (such as support/communication/monitoring/connectedness/involvement/social capital) and/or on various actors (i.e. parents/teachers/peers/classmates/neighbourhoods) of the social milieu. Despite their differences, these few studies consent to the salient role of each social setting in adolescents’ SHC, giving prominence to the family environment. Against this background, and based on the scarcity of relevant research evidence from Greece [9,24], our purpose was to bring together the family, school and peer context and investigate their associations with SHC in a large, nation-wide, school-based, adolescent sample in Greece.

Specifically, we aimed to focus on how adolescents perceive their well-being and functioning at home, at school and within their peer group and explore the extent to which these subjective perceptions predicted SHC. Sex and age in conjunction with adolescents’ subjective perceptions of the quality of their financial resources, which was used as a subjective indicator of economic status (subjective ES), were also assessed for their contributions in SHC and their effects were controlled for in further analysis. We hypothesized that adolescents’ perceived well-being in their family and school setting as well as among peers would each contribute a unique proportion of variance over and beyond that attributed to sex, age and subjective financial status. What is more, we hypothesized that the explained variance in SHC would be accumulated from each context.

## Methods

### Participants & procedure

The study was conducted in 2003 within the framework of the European project “Screening and Promotion for Health Related Quality of Life (HRQoL) in Children and Adolescents: A European Public Health Perspective” (acronym: KIDSCREEN) [25]. The school sampling in Greece was random, multi-staged and based on the age and sex distribution of school children living in the 54 geographical sectors of the country, according to data from the National Census of 2001. Schools in each sector were randomly selected by a computer program and students of each selected school were selected randomly from classroom name lists. A sample of 1.900 adolescents (12 to 18 year olds) was recruited. Inclusion criteria for students were: to belong in the age group under study, to be able to read and complete the questionnaire themselves and to consent to take part in the study. Students were asked to complete the questionnaire at school. Ethical approval was attained from the National Ministry of Education. Previous research on the representativeness of the present sample has reported that non-responder interviews showed no significant differences between responders and non-responders with regard to adolescents’ and parents’ general perceived health, parents’ marital status and highest educational level, and type of residence, indicating that a selection bias is less likely [26]. A total of 1.194 (63% response rate) of self-reported questionnaires were finally returned and 1.087 of them with full data were analysed.

### Measures

All predictor variables as well as the subjective indicator of economic status were part of the KIDSCREEN-52 instrument, a generic self-reported questionnaire for children and adolescents from 8 to 18 years with good psychometric properties [25]. In the present study, the adolescent version of the KIDSCREEN-52 instrument was used, tailored to children 12–18 years old. It is composed of 10 dimensions aiming to assess Health Related Quality of Life (HRQoL) from the adolescent’s perspective, to focus on physical, mental and social dimensions of well-being and to identify adolescents at risk with regard to their subjective health. It includes ten HRQoL dimensions: 1) physical well-being; 2) psychological well-being; 3) moods and emotions; 4) self-perception; 5) autonomy; 6) parent relations and home life; 7) social support and peers; 8) school environment; 9) social acceptance and bullying; and 10) financial resources. The KIDSCREEN-52 HRQoL questionnaire assesses either the frequency of behaviour/feelings or, in fewer cases, the intensity of an attitude. Both possible item formats use a 1 week recall period and a 5-point Likert response scale (1 = not at all/ never, 5 = excessively/always). Total score from each dimension ranges from 0 to 100, with higher scores indicating higher HRQoL. The Greek version of the instrument has been found to have good reliability [27] with Cronbach’s α for its 10 dimensions ranging satisfactorily between 0.73 (Bullying) - 0.90 (Moods & Emotion, Social Support & Peers). In the present study the dimensions of Parent Relations and Home Life, School Environment and Social Support and Peers (as predictor variables), as well as Financial Resources (as a background variable) of the KIDSCREEN-52 adolescent version were included in order to reflect adolescents’ perceived well-being in the family, school, peer and financial domain.

### Predictor variables

#### Family context

Adolescents’ perceived well-being in the family setting was assessed with the Parent Relations and Home Life dimension of the KIDSCREEN-52 instrument (α = 0.89). It comprises of 9 items that examine the relationship with the parents and the atmosphere in the adolescent’s home. It explores the quality of the interaction between adolescent and parent or carer, the adolescent’s feelings towards parents/carers, whether the adolescent feels loved and supported by the family, whether the atmosphere at home is comfortable or not and also if the adolescent feels treated fairly. Low scores on the Parent Relation & Home Life dimension indicate that the adolescent feels alone, overlooked, not appreciated and perceives parents as unavailable or unfair, whereas high scores reveal that the adolescent feels secure, supported, loved, well-understood and well cared-for and perceives parents as available and fair.

#### School context

Adolescents’ perceived well-being at school was explored by the School Environment dimension of the KIDSCREEN-52 instrument (α = 0.88). It includes 11 items examining adolescents’ perceptions and satisfaction with their cognitive capacity, concentration and performance at school. In addition, the dimension explores general feelings about school -such as whether school is an enjoyable place to be- and children’s view of their relationship with teachers, i.e. whether adolescents get along well with their teachers and whether teachers are perceived as being interested in the student as a person. Low scores demonstrate that the individual dislikes school and/or teachers, does not perform well and has negative feelings about school. High scores show that the adolescent performs well, feels happy at school and enjoys school life.

#### Peer context

Adolescents’ perceived well-being in their peer group was assessed with the Social Support and Peers dimension of the KIDSCREEN-52 instrument (α = 0.90). It includes 10 items considering social relations with friends and peers. The questions examine: the extent to which the adolescent feels accepted and supported by friends, adolescent’s ability to form and maintain friendships, aspects concerning communication with others, the extent to which the person experiences positive group feelings and how much he/she feels part of a group and respected by peers and friends. Low scores on the Social Support & Peers dimension indicate that the adolescent feels excluded, not accepted and supported by peers and unable to rely on them, while high scores show that the individual feels supported by and included in the peer group.

#### Dependent variable

##### Subjective health complaints

SHC were measured through the Health Behaviour in School-aged Children Symptom Checklist (HBSC-SCL; [28]), a self-administered brief screening instrument which indicates the frequency of occurrence of eight common health complaints. Students were asked: “In the last 6 months how often have you had the following?” and the items included were: headache, stomachache, backache, depressed mood, irritability, nervousness, sleeping difficulties, dizziness. Each health complaint was rated on a five-point frequency scale from “rarely or never”(1) to “about every day”(5). Items were added together to generate an index of psychosomatic health complaints score with minimum value = 1 and maximum value = 40. In quantitative analysis the HBSC-SCL has revealed a satisfactory reliability with test-retest reliabilities ranging from 0.70 to 0.80 [29]. In the present study, reliability analysis of the variables-components of SHC score was performed using Cronbach’s α coefficient and was found to be acceptable (α =0.78).

##### Background variables

Students were asked to report whether they are female (0) or male (1) and their age, which was coded in 2 categories (12-15 = 0 and 16-18 = 1).

Adolescents’ perceived quality of available financial resources (PQFR) was used as a subjective economic status indicator and was evaluated via the Financial Resources dimension of the KIDSCREEN-52 instrument (α = 0.89). The dimension includes 3 items that explore whether the adolescent feels that he/she has enough financial resources to allow him/her to live a lifestyle which is comparable to other children/adolescents and provides the opportunity to do things together with peers. Low scores indicate that they feel financially disadvantaged and that their lifestyle is restricted by their finances. High scores, on the contrary, show that they feel well-off and that they are satisfied with and enjoy their financial resources.

##### Statistical analysis

Continuous variables are presented as mean and standard deviation, whereas nominal variables are presented as absolute and relative frequencies. For bivariate correlations, Pearson’s r was used for the continuous variables and Spearman’s rho for the categorical variables. Data were modelled using multiple linear regression analysis with SHC as the outcome variable. In order to examine the unique contribution of perceived well-being in each context (family, school and peers) in the explanation of SHC, a hierarchical method of variable entry was selected. The background variables (sex, age and PQFR) were entered in step 1, in order to control for their effects in the following steps; the indicator for perceived well-being in the family setting was added in step 2; perceived school well-being was entered at step 3; finally, perceived well-being among peers was added in step 4, since the peer context is the social setting less explored in comparison to family and school, at least in Greece. The Durbin-Watson test was used to assess the assumption of independent errors. The data were also checked for multicollinearity using two measures: the tolerance and the variation inflation factor (VIF). All *p* values reported are two-tailed. Statistical significance was set at 0.05 and analyses were conducted using SPSS statistical software (version 20.0).

## Results

Descriptive statistics and bivariate correlations for all variables are presented in Table 1. The sample consisted of 659 girls (60.6%) and 428 boys (39.4%). 66.7% of the total sample (63.8% of girls and 71% of boys) belonged to the age category of 12–15 years, and the remaining 33.3% were between 16–18 years old. The mean score for SHC in our sample was 17.3, indicating a relatively moderate level of SHC. Concerning adolescents’ perceived well-being, highest scores were reported in regards to peer and family context (70.53 and 70.23 respectively), followed by available financial resources (69.75). The lowest score was documented for perceived well-being in the school setting (64.13).

**Table 1:** Descriptive statistics and bivariate correlations among all variables

	**Range/Categories**	**n**	**%**	**M**	**SD**	**1**	**2**	**3**	**4**	**5**	**6**	**7**
**Gender**^**† ǂ**^	Girls	659	60.6	na	na	1.00						
	Boys	428	39.4									
**2. Age**^**‡ ǂ**^	12-15	725	66.7	na	na	−0.07*	1.0					
	16-18	362	33.3									
**3. PQFR**	0-100	na	na	69.75	24.25	0.05	−0.06**	1.00				
**4. Family context**	0-100	na	na	70.23	20.21	0.11*	−0.18*	0.33*	1.00			
**5. School context**	0-100	na	na	64.13	18.58	−0.003	−0.27*	0.27*	0.47*	1.00		
**6. Peer context**	0-100	na	na	70.53	21.17	0.06	−0.11*	0.24*	0.30*	0.19*	1.00	
**7. SHC**	1-40	na	na	17.3	0.78	−0.22*	0.18*	−0.21 *	−0.4 *	−0.33*	−0. 25*	1.00

M = arithmetic mean, SD = standard deviation, PQFR = Perceived Quality of Financial Resources, SHC = subjective health complaints.

^†^Girls were coded as 0 and boys as 1.

^‡^ Age category 12–15 was coded as 0 and age category 16–18 was coded as 1.

^ǂ^ Spearman correlation coefficient.

*p < 0.01, **p < 0.05.

Moderate correlations (Pearson’s r > 0.30) were observed between perceived family well-being and SHC, PQFR and perceived school well-being as well as between SHC and perceived school well-being. Before the hierarchical multiple regression analysis was performed, background and predictor variables were examined for collinearity. VIF and tolerance values ranged from 1.007 to 1.45 and from 0.69 to 0.99 respectively, suggesting that the estimated *β*s are well established in the following regression model.

Table 2 summarizes the results of the hierarchical regression analysis. Background variables (sex, age and PQFR) entered in Step 1 accounted for 10.6% of the total variation in SHC. SHC levels were lower for boys (*β = −0.186, p < 0.001)* and higher for older adolescents (*β = 0.142, p < 0.05).* PQFR was shown to have a small contribution on SHC in Step 1 (*β = −0.199, p < 0.001).* Perceived family well-being entered at Step 2 explained an additional 9.3% of the variation in SHC *(f change = 126 393, p < 0.001)*, increasing the proportion of variance explained by the model to 20%. Higher degrees of perceived family well-being were associated with lower levels of SHC (*β = −0.332, p < 0.001).* Perceived school well-being entered at Step 3 accounted for an additional 2.7% of the variation in SHC *(f change = 37 175, p < 0.001)*, with a higher degree of perceived school well-being being associated with lower levels of SHC (*β = −0.19, p < 0.001).* Finally, perceived well-being among peers was entered at Step 4 and accounted for an additional 1.3% of the variation in SHC (*f* change *= 18 501, p < 0.001*). Higher degrees of perceived well-being among peers were found to associate with lower levels of SHC (*β = −0.122, p < 0.001).* All variables remained highly significant (*p < 0.001)* at Step 4, except for age *(p < 0.05)* and PQFR *(p = 0.101)*.

**Table 2:** Summary of hierarchical multiple linear regression analysis for variables predicting adolescents’ SHC

**Variables**	**SHC**			
	β† Step 1	β† Step 2	β† Step 3	β† Step 4
Step 1: Background variables				
Gender‡	-.186*	-.156*	-.172*	-.167*
Ageǂ	.142*	.094*	.059***	.055***
PQFR	-.199*	-.090**	-.066***	-.047
Step 2: Perceived family wellbeing				
Perceived family wellbeing		-.332*	-.256*	-.228*
Step 3: Perceived school wellbeing				
Perceived school wellbeing			-.190*	-.186*
Step 4: Perceived wellbeing among Peers				
Perceived wellbeing among Peers				-.122*
Model summary				
ΔF	42.98*	126.39*	37.17*	18.50*
Df	3, 1083	1, 1082	1, 1081	1, 1080
R^2^	.106	.200	.226	.239
ΔR^2^	.106	.093	.027	.013

β† = standardized regression coefficients.

‡Girls were coded as 0 and boys as 1.

ǂ Age category 12–15 was coded as 0 and 16–18 as 1.

*p < 0.001 **p < 0.005 ***p < 0.05.

In all cases, effect sizes were small [30]. The regression models were statistically significant and explained a total of approximately 24% of the variance in SHC. The Durbin-Watson value (1.982) indicated that the assumption of independent errors was almost certainly met.

## Discussion

The aim of the present study was to investigate the extent to which Greek adolescents’ perceived well-being in three main social contexts (family, school and peers) predicted SHC. We employed a hierarchical regression model in order to i) control for the effects of previously well-established demographic factors (sex, age and subjective economic status) and ii) to identify the unique proportion of variance attributed to each context. As hypothesized, adolescents’ perceived well-being in each of the three social contexts appeared to hold unique proportions of variance in self-reported SHC, after controlling for the effects of sex, age and subjective economic status. In addition, our final model confirmed that the explained variance in SHC was accumulated from each social context studied.

### Demographic & economic factors

Consistent with ample research evidence [19,22,31], female sex and older age were found to be significantly associated with SHC. In conjunction with subjective economic status, these three factors were found to account for the largest amount of variance in SHC. The employment of a subjective type of assessment of financial resources, instead of more “objective” indicators such as the Family Affluence Scale or parental educational or employment status, was justified by the overall orientation of our study on adolescents’ perceived well-being and functioning. In addition, our decision was bolstered by current research evidence supporting that subjective measures of financial/social status constitute significant correlates of adolescent health outcomes [9,32,33]. In our previous study, adolescents’ PQFR was found to have a genuine but small impact on self-reported SHC [31]. Given that adolescence is a period when self-conceptualization matures, the individual’s subjective sense of his/her financial standing may reflect aspects of self-perceived social comparability and could potentially become a source of distress, thus, affecting adolescent subjective health [31,32].

### Perceived well-being in the family context

The family setting, as the primary social unit in children’s lives, appeared to retain its salience throughout adolescent years. Perceived family well-being reflected adolescents’ perceptions of their relationships with parents and the overall atmosphere at home. It was identified as a substantial determinant of adolescents’ SHC by demonstrating the highest individual contribution among all factors under study in terms of both the standardized regression coefficient (standardized β) and the R^2^change. Our finding is in the same line with previous research that underlines the family environment for its prominent role in adolescent psychosomatic health [19,22]. Even though the quest for autonomy and independence from parents is an indispensable aspect of adolescent psychosocial development, maintaining supportive and secure family relationships and enjoying a cohesive family atmosphere are of paramount importance for adolescent psychosomatic adjustment [2-4].

Albeit, it should be stressed that the extreme ends of the parental support spectrum, reflected by emotionally deprived and neglectful family backgrounds or by anxious, over-protective, emotionally over-involved parenting have both been associated with the development, increase and maintenance of SHC and various unexplained pain symptoms in childhood and adolescence [3,34,35]. A similar finding has been documented regarding families with a rigid orientation to child’s compliance and high achievements [3,35]. On the contrary, a secure home environment, where the child feels respected, supported and well-treated, is not only linked to positive health outcomes, but has also been suggested to buffer against stressful life events and to enhance social competence [8,36]. Our finding could have important implications in health promotion planning in community and healthcare settings, so as family-tailored initiatives aiming to enhance parent–child relations and educate parents in smoothing out family functioning during adolescence are properly implemented.

### Perceived well-being in the school context

Perceived well-being in the school context was found to contribute a modest amount of variance in adolescents’ self-reported SHC, over and above that attributed to sex, age, PQFR and perceived family well-being. Even though our finding is modest, it corroborates previous study findings [10,37,38] as well as the study of Karademas et al. [9] who reported a maximum of 15% amount of variance in SHC explained by both family and school factors throughout adolescent years. In our study, perceived school well-being focused on adolescents’ perceptions of their cognitive capacities, learning and concentration, as well as on their feelings about their teachers and school, avoiding any overlap with peer relations that were examined separately. Relevant school-related factors, such as academic achievement, schoolwork load and relationships with teachers have been consistently reported to constitute strong determinants of SHC in adolescence [2,10,14].

However, it should be stressed that the mean score of perceived school well-being reported by the participants in our study was rather moderate (Table 1). More importantly, it was the lowest in comparison with the mean scores of adolescents’ subjective assessments of their well-being and functioning in the family, peer and financial domain. This may indicate that adolescents are expected to spend increasing amounts of time in a school environment that they do not feel adequately connected with or satisfied with the overall atmosphere, their relationships with teachers and their own functioning. With this in mind, our finding that lower degrees of perceived school well-being are associated with higher levels of SHC could depict a rather adverse impact of the school setting on adolescent psychosomatic health. This is an alarming outcome, given the central role of the school environment in adolescents’ lives [16,38]. Policymakers should be attentive to detect and improve those aspects of school life that constitute sources of distress for students. In Greece, particularly, it is of paramount importance that the highly competitive school climates, that lay great emphasis on academic performance, are re-oriented towards a more inclusive and collaborative school culture. Implementation of school mental health promotion initiatives that aim to foster students’ psychosocial resources and skills could be effective in enhancing a pro-social school orientation.

### Perceived well-being in the peer context

Above and beyond background variables and perceived family and school well-being, adolescents’ perceptions of the nature and quality of their relationships with peers held a unique and salient role in explaining SHC variance. What is more, the mean score of adolescents’ perceived well-being among peers was the highest in relation to the respective scores in the family, school and financial domain, indicating that Greek adolescents seem to feel rather comfortable in their peer group. During adolescent years, the peer group emerges as a powerful socializing agent that provides an arena where self-identities are explored and social skills are tested and transformed [39]. Peer affiliations have been highlighted for their profoundness in psychosocial development, as wellsprings of social comparison and identification in the process of individuation and identity formation.

Our finding indicated that the peer context held a small but unique role in adolescent psychosomatic health. Given that peer relations gain increasing importance as children grow older [40], the small contribution reported in our study could be attributed to the overrepresentation of younger adolescents in our sample. Being unpopular or socially isolated has been associated with adverse outcomes in psychosomatic health and psychosocial adjustment [5,23,41]. More importantly, though, recent research suggests that difficulties in forming and maintaining reciprocal relationships with peers could be represented by a psychosomatic health gradient not only in adolescence, but also in adulthood [42]. Therefore, promoting opportunities for and fostering supportive peer interactions in community and school settings could have a protective effect on psychosomatic health outcomes in adolescence that may well extend to adult years.

### Strengths and limitations

Our study sheds light on the unique and cumulative contributions of adolescents’ perceived well-being in the family, school and peer setting in the explanation of SHC. Recruitment of a nation-wide, random, school sample and the use of standardized, subjective well-being tools lend to our study extra merit. However, the cross-sectional design employed does not permit any causal relationships to be inferred. In discussing study limitations, some sampling issues should be acknowledged. There was a tendency in our sample for a higher response rate from girls compared with boys and from younger participants in relation to older ones. Even though this tendency is common in school-based surveys and across sampling methods and countries, caution is required, since school-based surveys do not necessarily provide the most representative samples, at least in terms of age and sex. In the context of the KIDSCREEN study, however, further procedures were implemented in order to assess the representativeness of national samples. National samples were compared with the corresponding reference population in the Eurostat census database in terms of age, sex and highest educational level for men and women and additional analyses were performed using re-sampling methods, such as bootstrapping. These showed that the KIDSCREEN survey is sufficiently representative when it comes to providing reference population values [26]. In addition, no other informant was involved, making our data susceptible to biased estimates. Another point of criticism is that the main purpose of the KIDSCREEN study was to assess children’s and adolescents’ HRQoL via the standardized KIDSCREEN instrument, not to evaluate determinants of children’s and adolescents’ SHC. Therefore, the findings of the present study should be considered preliminary, since they derive from secondary use of data primarily collected for other means. Moreover, a thorough investigation of adolescents’ SHC was also hampered by non-inclusion of other factors that have been previously supported to influence self-reported SHC during adolescence, such as adolescents’ chronic conditions [8], sense of coherence [1], and neighbourhood social capital [22]. Future research should address these limitations and perhaps elucidate potential sex differences that have been suggested by some studies [8,13].

## Conclusions

Despite of the aforementioned caveats, our findings highlight potentially important avenues in the comprehension of SHC development and maintenance in adolescence. Perceived well-being in the family, school and peer context appears to hold unique as well as cumulative contributions to self-reported SHC. Our study adds to the limited research on the field, since it includes and underscores the contribution of peer interactions, as a separate social milieu, in concert with family and school-related factors. Deeper understanding of how adolescents perceive and feel in their social contexts and the relationship with their psychosomatic health could lead to a comprehensive framework for adolescents’ SHC. Our study suggests that efforts to both remedy SHC, as well as promote psychosomatic health and adjustment through adolescent years, need to include multiple salient contexts. The social component of adolescents’ experiences should be an indispensable part of the “routine” assessment of an individual presenting with SHC in school or community healthcare settings. Timely referrals to counseling services could help families overcome communicational barriers and establish clear and cohesive relationships during the challenging adolescent period, with potential positive implications in adolescent psychosomatic health. In tandem, implementation of mental health promotion initiatives in schools, that focus on inclusiveness and foster peer collaboration and social competencies, could improve school culture, enhance adolescents’ connectedness with teachers and school and promote intimate relationships among peers. A high-quality social network in the family, school and peer setting could contribute individually and in concert in navigating the individual smoothly across the multiple changes and emerging challenges of the adolescent period to adulthood. As the complexities of the relational web surrounding adolescent psychosomatic ill-health are unraveled, a multi- and inter-disciplinary approach to adolescent psychosomatic development becomes of paramount importance.

## Abbreviations

SHC: Subjective health complaints; PQFR: Perceived quality of financial resources; HRQoL: Health related quality of life; HBSC-SCL: Health behaviour in school-aged children symptom checklist; subjective ES: Subjective economic status; VIF: Variation inflation factor.

## Competing interests

The authors declare that they have no competing interests.

## Authors’ contributions

DP conceived of the study and carried out the writing of the manuscript. ED performed the statistical analysis. CD and GK participated in revising the paper. YT had overall supervision of the study. All authors read and approved the final manuscript.
